# Expression of HIF-1α and CAIX in nasopharyngeal carcinoma and their correlation with patients’ prognosis

**DOI:** 10.1007/s12032-014-0304-1

**Published:** 2014-11-07

**Authors:** Yuhan Chen, Xianming Li, Shihai Wu, Gang Xu, Yayan Zhou, Long Gong, Zihuang Li, Dong Yang

**Affiliations:** Department of Radiation Oncology, Second Clinical Medicine College of Jinan University, Shenzhen, 518020 China

**Keywords:** Nasopharyngeal carcinoma, Hypoxia-inducible factor-1 alpha, Carbonic anhydrase IX, Prognosis

## Abstract

This study investigates the expression of hypoxia-inducible factor-l alpha (HIF-1α) and carbonic anhydrase IX (CAIX) in nasopharyngeal carcinoma (NPC) tissues and their correlation with clinicopathological features and prognosis in NPC patients. The expression of HIF-1α and CAIX proteins was detected by immunohistochemical staining in 129 samples of NPC and 20 samples of chronic nasopharyngitis. The correlations between the expression of these two proteins and clinicopathological features and prognosis were evaluated in NPC patients. Our results showed that the positive expression rates of HIF-1α and CAIX proteins in NPC were significantly higher than those in chronic nasopharyngitis (both *P* < 0.01). In addition, high HIF-1α protein expression was correlated with lymph node metastasis and advanced clinical stage for NPC patients (both *P* < 0.01), whereas there were no findings of correlations between CAIX protein expression and gender, age, T stage, node involvement and clinical stage (all *P* > 0.05). The Spearman analysis indicated that HIF-1α was positively correlated with CAIX expression (*r* = 0.249, *P* = 0.004). HIF-1α and CAIX co-expression was associated with the poor overall survival (OS), progression-free survival (PFS), loco-regional relapse-free survival (LRRFS) and distant metastasis-free survival (DMFS) in NPC patients (*P* = 0.017, *P* = 0.022, *P* = 0.033, and *P* = 0.017, respectively). Multivariate analysis showed that the positive expression of CAIX protein was an independent prognostic factor for PFS, LRRFS and DMFS. In conclusion, overexpression of HIF-1α and CAIX might be involved in the carcinogenesis and development of NPC and they were associated with patients’ poor prognosis.

## Introduction

Nasopharyngeal carcinoma (NPC) is one of the most common malignancies in China and Southeast Asia areas [1]. With the development of radiotherapy technology and the combined application of radiochemistry, the therapeutic efficacy is gradually improved, but it still fails to achieve the desired effect because of local recurrence and distant metastasis [2].

Hypoxia is one of the most common characteristics in many solid cancers. Tumor hypoxia is known to be mainly responsible for tumor resistance to radiotherapy and chemotherapy as well as to promote tumor phenotype influencing invasiveness, metastasis and poor prognosis [3]. There is now evidence to indicate that cells triggering an adaptive response to hypoxia conditions are mediated by hypoxia-inducible factor-1 (HIF-1)-dependent pathway in cancer. HIF-1 is a heterodimeric basic helix-loop-helix transcription factor consisting of HIF-1α and HIF-1β subunits. The biological function of HIF-1 is mainly determined by the expression and activity of HIF-1α [4]. HIF-1α plays a major role in several aspects of tumor biology, such as glucose uptake, metabolism, growth rate, angiogenesis, invasiveness, metastasis and apoptosis [5]. Overexpression of HIF-1α is common in many malignancies and has been found to be correlated with a poor prognosis of different types of tumor. Kitagawa et al. [6] reported that the NPC patients with overexpression of HIF-1α had significant worse prognosis.

Under hypoxic conditions, HIF-1 is activated and induces the up-regulation and overexpression of a variety of genes. Among these genes, carbonic anhydrase IX (CAIX) is significantly over expressed in a variety of malignancies [7]. CAIX is an isomeric member of the CA family, whose main function is to catalyze the reversible reaction of CO_2_ hydration, involved in the maintenance of normal intracellular pH value and regulation of extracellular acidic microenvironment formation enhancing tumor cell growth, invasiveness and migration [8]. It indicates that CAIX plays an important role in the development and progression of malignant tumor. Several findings have showed that CAIX expression is related to poor prognosis [9, 10]. Although CAIX alone had not showed any prognostic effect for NPC patient outcome, previous study have confirmed that CAIX predicts poor prognosis for other types of head and neck cancer, such as laryngeal carcinoma [11].

Therefore, it is essential to identify the biological markers associated with the diagnostic and prognostic features of NPC. In this study, we identified the expression of HIF-1α and CAIX in NPC patients and assessed their correlations with clinicopathological features and prognosis of patients.

## Materials and methods

### Patients and tumor biopsies

We obtained 129 tumor specimens from NPC patients who had been diagnosed at the second clinical medicine college of Jinan University, China, from December 2006 to August 2011. The tumor biopsies were taken prior to patients received chemotherapy or radiotherapy at the time. Twenty samples of chronic nasopharyngitis were used as controls. Of 129 patients, there were 99 men and 30 women, with a median age of 43 years (range 20–77). The patients’ characteristics are shown in Table 1. Clinical status was determined according to the 2010 American Joint Committee on Cancer (AJCC, 7th edition). All patients were received either standard curative radiotherapy with or without chemotherapy. The last follow-up date was at the end of December 2013, and the median time of follow-up was 52.4 months (range 8.6–84.1). The current project was approved by the local ethics committee, and informed consent was obtained from all patients.

**Table 1:** Patient characteristics

Characteristic	No. of patients (%)
Patients	129 (100)
Gender	
Male	99 (76.7)
Female	30 (23.3)
Age (years)	
<50	88 (68.2)
≥50	41 (31.8)
T classification	
T1	33 (25.6)
T2	46 (35.7)
T3	28 (21.7)
T4	22 (17.0)
N classification	
N_0_	9 (6.9)
N_1_	34 (26.4)
N_2_	65 (50.4)
N_3_	21 (16.3)
Clinical stage	
I	2 (1.5)
II	28 (21.7)
III	58 (45.0)
IV	41 (31.8)
KPS	
60	1 (0.8)
70	14 (10.8)
80	57 (44.2)
90	57 (44.2)
Treatment arm	
RT alone	53 (41.1)
Radiochemistry	76 (58.9)

### Immunohistochemical staining and evaluation

Immunohistochemical staining of HIF-1α and CAIX expressions was performed on 4-μm sections from NPC paraffin-embedded tissues. After de-paraffinisation and hydration, antigen retrieval was performed by pressure cooking for 20 min in citric acid buffer. The sections were treated with 3 % hydrogen peroxide for 10 min to block endogenous peroxidase activity. Each section was incubated with protein blocker for 15 min at room temperature followed by mouse anti-human HIF-1α antibody(1:100, Novus Biologicals, Colorado, USA) and rabbit anti-human CAIX antibody (1:400, Novus Biologicals, Colorado, USA)incubation at 4 °C overnight. After washing with PBS, the sections were incubated with secondary antibody for 20 min at room temperature. The reaction products were developed using diamidobenzidine tetrahydrochloride (DAB) solution (Cell Signaling Technology, CA, USA) for 10 min. The sections were counterstained with hematoxylin.

Using PBS instead of primary antibody as negative control was conducted for each staining. Known positive results from the prepared experiments were used as positive control. For each slide, five arbitrarily separated microscopic fields (400×) with every field containing >100 tumor cells were evaluated by two pathologists blinded to the clinical data. The brown granules in nuclear membrane were considered as positive staining for HIF-1α while in cell membranes or cytoplasm for CAIX. Staining intensity was scored as follows: 0, no staining; 1, weak staining; 2, moderate staining; and 3, strong staining. Scores for the percentage of immunopositive cells were rated as follows: 0, <10 % positive cells; 1, 10–25 %; 2, 25–50 %; and 3, >50 % positive cells. Taking the scores of intensity of staining and percentage of immunopositive cells together, the cases were classified into negative group with scores ≤2 and positive group with scores >2.

### Statistical analysis

The clinical characteristics of patients in relation to HIF-1α and CAIX expressions were analyzed using the Chi-square test. The Spearman correlation test was used to analyze the correlation between HIF-1α and CAIX expressions in NPC. Association of expression of HIF-1α and CAIX with overall survival, progression-free survival, loco-regional relapse-free and distant metastasis-free survival rates were determined in Kaplan–Meier analyses and log-rank test. The Cox regression analysis was used for multivariant analysis. *P* < 0.05 was considered statistically significant. Statistical analysis was carried out using SPSS version 16.0 (SPSS, Chicago, IL, USA). Overall survival (OS) was defined as the time from the start of radiotherapy to the date of death or last follow-up. Progression-free survival (PFS) was defined as the time from the start of radiotherapy to the date of progression (loco-regional relapse or distant metastasis) or last follow-up. Loco-regional relapse-free survival (LRRFS) was defined as the time from the start of radiotherapy to the date of loco-regional relapse or last follow-up. Distant metastasis-free survival (DMFS) was defined as the time from the start of radiotherapy to the date of distant metastases or last follow-up.

## Results

### Expression of HIF-1α and CAIX in NPC and chronic nasopharyngitis

**Fig. 1:**
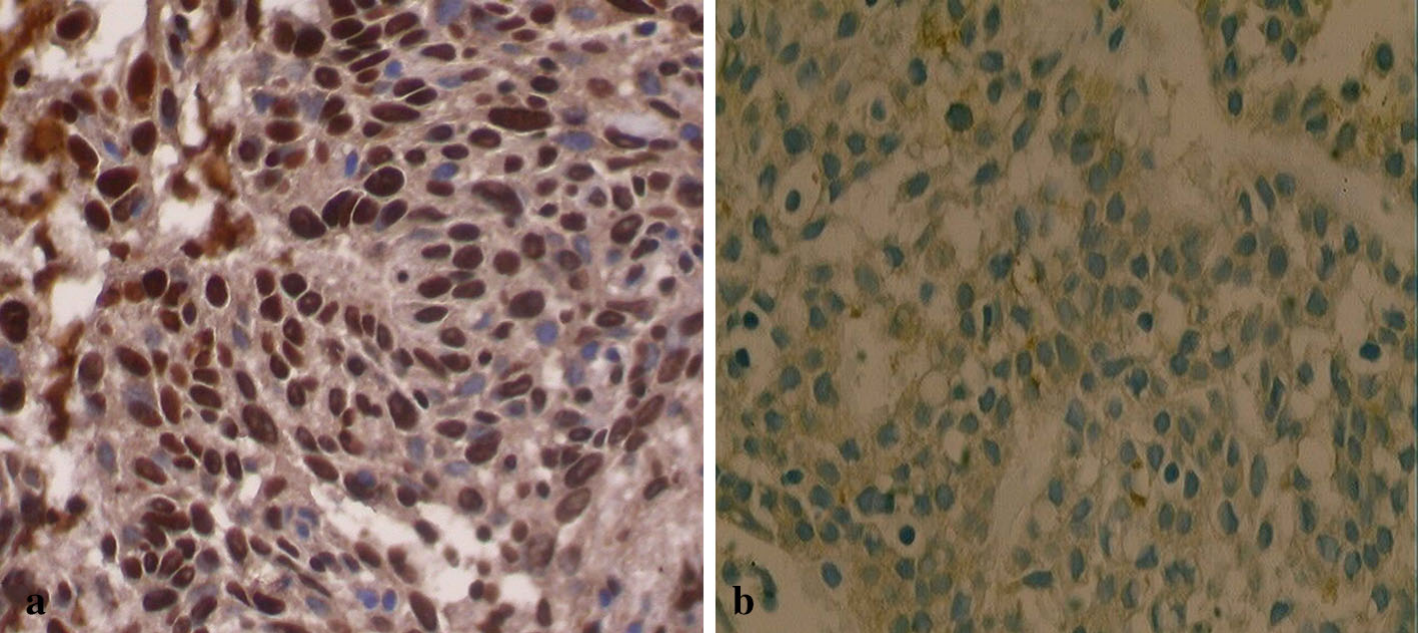
Expression of HIF-1α and CAIX by immunohistochemistry in NPC tissues (original magnification ×400). **a** Nuclear membrane positive staining of HIF-1α in NPC tissue. **b** Plasmalemma and cytoplasmic positive staining of CAIX in NPC tissue

The expression of HIF-1α and CAIX was examined by immunohistochemical staining in NPC biopsy specimens (Fig. 1). Of 129 NPC biopsy sections, positive expression of HIF-1α and CAIX was observed in 53.5 % (69/129) and 56.6 % (73/129) of NPC samples, respectively. While in 20 samples of chronic nasopharyngitis, no positive expression of HIF-1α was detected and there was 15 % (3/20) of sections with CAIX positive expression. The positive rates of HIF-1α and CAIX expressions in NPC were significantly higher than chronic nasopharyngitis (*χ* ^2^ = 19.924, *P* < 0.01; *χ* ^2^ = 11.985, *P* < 0.01). The Spearman analysis revealed that HIF-1α expression was positively correlated with CAIX expression (*r* = 0.249, *P* = 0.004).

### Relationship between expression of HIF-1α and CAIX proteins and clinicopathological features

HIF-1α expression was significantly correlated with lymph node stage and clinical stage, but there was no significant association between HIF-1α expression and age, gender and T stage. There was no relationship between CAIX expressions with any of the following clinical parameters: age, gender, primary tumor stage, lymph node stage and clinical stage (Table 2).

**Table 2:** Correlation between expression of HIF-1α and CAIX and clinicopathological features

Variables	Total	HIF-1α		*P* value	CAIX		*P* value
		Positive (%)	Negative (%)		Positive (%)	Negative (%)	
Gender							
Male	99	53 (53.5)	46 (46.5)	0.984	57 (57.6)	42 (42.4)	0.681
Female	30	16 (53.3)	14 (46.7)		16 (53.3)	14 (46.7)	
Age(years)							
<50	88	42 (47.7)	46 (52.3)	0.055	48 (54.5)	40 (45.5)	0.493
≥50	41	27 (65.9)	14 (34.1)		25 (61.0)	16 (39.0)	
T classification							
T_1+2_	79	39 (49.4)	40 (50.6)	0.238	43 (54.4)	36 (45.6)	0.534
T_3+4_	50	30 (60.0)	20 (40.0)		30 (60.0)	20 (40.0)	
N classification							
N_0_	9	1 (11.1)	8 (88.9)	**0.008**	3 (33.3)	6 (66.7)	0.144
N_1–3_	120	68 (56.7)	52 (43.3)		70 (58.3)	50 (41.7)	
Clinical stage							
I + II	30	7 (23.3)	23 (76.7)	**0.000**	13 (43.3)	17 (56.7)	0.094
III + IV	99	62 (62.6)	37 (37.4)		60 (60.6)	39 (39.4)	

Significant results are given in bold

### Prognosis analysis

**Fig. 2:**
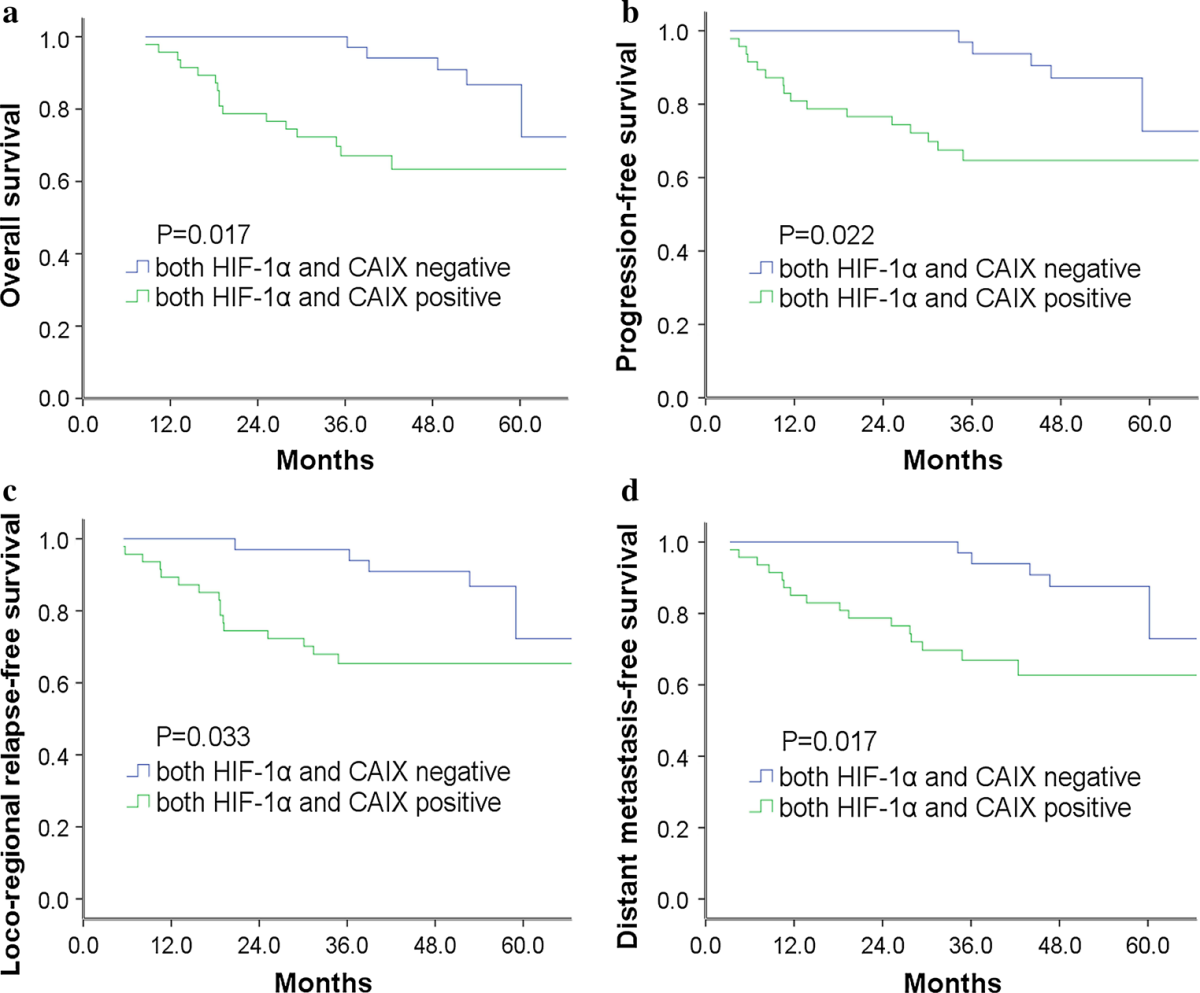
The Kaplan–Meier curves for NPC patients with both positive and negative expressions of HIF-1α and CAIX: **a** overall survival, **b** progression-free survival, **c** loco-regional relapse-free survival, **d** distant metastasis-free survival

To the date of last follow-up, there were 18 patients with loco-regional relapse, 20 patients with distant metastasis and 29 deaths. NPC patients’ 5-year OS, PFS, LRRFS and DMFS with respect to HIF-1α and CAIX status are summarized in Table 3. NPC patients with positive expression of HIF-1α had poor OS and DMFS than those with negative HIF-1α expression. However, HIF-1α expression had no influence on the PFS and LRRFS. For the survival of CAIX, CAIX positive cases had worse OS, PFS, LRRFS and DMFS than CAIX negative cases. Kaplan–Meier survival curves showed that patients with both HIF-1α and CAIX positive expressions were associated with poorer OS, PFS, LRRFS and DMFS than those with both HIF-1α and CAIX negative expressions (Fig. 2). Moreover, a univariate analysis by Kaplan–Meier method also showed that age, Karnofsky Performance Scale (KPS), node involvement and clinical stage were associated with a poor prognosis (*P* < 0.05). Finally, a multivariate Cox regression analysis revealed that significantly independent prognostic factors for OS were age and clinical stage. Significantly independent prognostic factors for PFS, LRRFS and DMFS were age, KPS and the expression of CAIX protein (Table 4).

**Table 3:** Association of HIF-1α and CAIX expressions with 5-year survival in NPC patients

Group	5-year OS		5-year PFS		5-year LRRFS		5-year DMFS	
	Rate (%)	*P* value	Rate (%)	*P* value	Rate (%)	*P* value	Rate (%)	*P* value
HIF-1α								
Positive	71.5	**0.023**	73.0	0.074	72.4	0.072	72.4	**0.030**
Negative	79.6		81.5		81.8		79.3	
CAIX								
Positive	68.5	**0.044**	67.3	**0.035**	67.9	**0.046**	67.5	**0.042**
Negative	76.7		77.4		76.8		77.6	
HIF-1α and CAIX								
Both positive	63.4	**0.017**	64.7	**0.022**	65.3	**0.033**	62.7	**0.017**
Both negative	72.3		72.6		72.3		73.0	

Significant results are given in bold

*OS* overall survival, *PFS* progression-free survival, *LRRFS* loco-regional relapse-free survival, *DMFS* distant metastasis-free survival

**Table 4:** The result of multivariate analysis of NPC patients after radiotherapy

Variables	*β* value	SE value	Chi-square value	*P* value	RR (95 % CI)
OS					
Age	0.065	0.017	14.727	0.000	1.067 (1.032–1.103)
Clinical stage	0.701	0.305	5.274	0.022	2.016 (1.108–3.666)
PFS					
Age	0.074	0.017	19.064	0.000	1.077 (1.042–1.114)
KPS	−0.786	0.276	8.093	0.004	0.456 (0.265–0.783)
CAIX	1.093	0.434	6.333	0.012	2.983 (1.273–6.987)
LRRFS					
Age	0.078	0.018	19.666	0.000	1.081 (1.044–1.118)
KPS	−0.728	0.265	7.574	0.006	0.483 (0.287–0.811)
CAIX	1.047	0.433	5.852	0.016	2.849 (1.220–6.653)
DMFS					
Age	0.078	0.017	20.170	0.000	1.081 (1.045–1.118)
KPS	−0.755	0.276	7.491	0.006	0.470 (0.274–0.807)
CAIX	1.016	0.432	5.542	0.019	2.762 (1.185–6.434)

*OS* overall survival, *PFS* progression-free survival, *LRRFS* loco-regional relapse-free survival, *DMFS* distant metastasis-free survival, *KPS* Karnofsky Performance Scale, *SE* standard error, *RR* risk ratio, *CI* confidence interval

## Discussion

In our study, we found that positive staining of HIF-1α was detected in 53.5 and 0 % of nasopharyngeal carcinoma cases and chronic nasopharyngitis tissues, respectively. This is in agreement with previous reports of HIF-1α which was over expressed in NPC [12–14]. Shou et al. [15] reported that HIF-1α expression was significantly associated with high T stage and lymph node metastasis. Our study showed that positive expression of HIF-1α protein was correlated with node involvement and clinical stage for NPC patients, but not with age, gender and T stage of NPC patients. Gong et al. [16] conducted a systematic review from 28 studies to assess the association between HIFs and head and neck cancer (HNC) and showed that overexpression of HIFs was significantly associated with the increase of mortality risk survival in HNC and worse OS in nasopharyngeal carcinoma. Moreover, other findings have showed that positive expression of HIF-1α was associated with a higher distant metastasis, a worse overall and disease-free survival in NPC patients [14]. Similar to their results, we found that patients with high expression of HIF-1α were significantly associated with worse OS and DMFS, but the level of HIF-1α expression had no effect on PFS and LRRFS. From these results, we speculated that HIF-1α may enhance cancer mortality by promoting distant metastasis. Moon et al. [17] reported that metastasis-associated protein 1 enhanced angiogenesis by stabilization of the HIF-1α transcription complex, which promoted tumor progression and metastasis. Yang et al. [18] showed that overexpression of HIF-1α not only promoted epithelial-mesenchymal transition and metastastic phenotypes, but also up-regulated the expression of the transcription factor TWIST, an essential mediator of cancer metastasis, resulting in treatment failure and mortality in malignancy. Jing et al. [19] found that hypoxia-induced invasion and metastasis of esophageal carcinoma was attributed to the distinctive capacity of HIF-1α in inhibiting E-cadherin and promoting matrix metalloproteinase-2 expression. The vascular endothelial growth factor, cyclooxygenase-2 and isocitrate dehydrogenase-2 were also found to be involved in the regulation of HIF-1α signaling pathway for cancer angiogenesis, invasiveness and metastasis [20–22].

We have found that CAIX was expressed in 56.6 % of NPC biopsy specimens, and no association was identified between the expression of CAIX and age, gender, tumor stage, nodal stage and clinical stage. Our observations were similar to those findings by other investigators in NPC [12]. These results suggest that CAIX probably involves in the occurrence and development of NPC during which it keeps an overexpression status. Peridis et al. [23] conducted random-effect meta-analytical techniques to evaluate the prognosis of head and neck cancer with CAIX expression from sixteen studies and found that the presence of CAIX in head and neck malignant tumors was associated with reduced overall survival and disease-free survival. Hui et al. [12] reported that there was no significant association of the expression of CAIX with local recurrences and distant metastases in NPC. However, our studies revealed that positive expression of CAIX was significantly associated with worse OS, PFS, LRRFS and DMFS in patients with NPC receiving irradiation with or without chemotherapy. Multivariate analysis using the Cox’s regression analysis showed that CAIX protein was an independent prognostic indicator for patients’ PFS, LRRFS and DMFS. It was found out that expression of the enzyme CAIX on the tumor cell surface was involved in pH regulation by hydrating cell-generated CO_2_ into HCO_3_^−^ and H^+^ facilitating the extracellular trapping of acid, which contributed to the acidification of the microenvironment favoring tumor growth, invasion and development [24]. In addition, CAIX had a capacity to reduce E-cadherin-mediated cell adhesion via interaction with beta-catenin, while loss or destabilization of E-cadherin, a key adhesion molecule, was correlated with tumor invasion [25]. Moreover, several important molecular regulators involved in cancer angiogenesis, apoptosis inhibition and cell–cell adhesion disruption, including epidermal growth factor receptor, c-erbB-2 and MUC1, had been found to be associated with the expression of CAIX, which might explain the relationship between CAIX and inferior prognoses [26].

Furthermore, we identified that the expression of HIF-1α was significantly correlated with the expression of CAIX. Similarly, Hui et al. [12] found that tumor expression of HIF-1α was strongly relevant to that of CAIX in 90 patients with NPC. The co-expression of HIF-1α with CAIX in cell lines and their regulatory mechanisms have been well documented. With regard to in vitro hypoxia experiment, Wykoff et al. [27] found that hypoxia-inducible activity of the CAIX promoter was mediated by HIF-1 pathway and further study demonstrated that CAIX promoter was tightly regulated by a HIF-1-dependent hypoxia response element (HRE) lying adjacent to the initiation site. Since HIF-1α up-regulates the expression of downstream genes CAIX, we considered HIF-1α and CAIX as endogenous hypoxia-related markers and found that both HIF-1α and CAIX positive expression were associated with poor prognosis of NPC. It is consistent with previous reports of overexpression of HIF-1α and CAIX correlating with a poor prognosis in several types of cancer, including NPC, laryngeal carcinoma, breast cancer and non-small cell lung cancer [28–30]. Our finding suggests that, besides primary radiotherapy, some other supplementary treatment, such as hypoxia-modifying and gene targeted therapy, should be considered for NPC patients showing high HIF-1α or CAIX expression.

In summary, overexpression of HIF-1α and CAIX can exploit synergies in carcinogenesis and development of NPC. HIF-1α high expression was correlated with node involvement, clinical stage and poor prognosis of NPC. CAIX high expression was relevant to poor prognosis and could serve as an independent prognostic factor in NPC. Radiotherapy combined with HIF-1α related CAIX signaling pathway targeted therapeutic approaches would be a possible solution to improve therapeutic efficiency for NPC.
